# Acute Rupture of the Plantar Fascia in a Soccer Player

**DOI:** 10.7759/cureus.38527

**Published:** 2023-05-04

**Authors:** Diogo Costa, Patrícia Cruz, Rui Brito, Pedro Cantista, Sérgio Rodrigues-Gomes

**Affiliations:** 1 Physical Medicine and Rehabilitation, Centro Hospitalar e Universitário do Porto, Porto, PRT; 2 Radiology, Espregueira-Mendes Sports Center, Porto, PRT

**Keywords:** athlete, soccer player, plantar fasciitis, acute rupture, plantar fascia

## Abstract

Acute rupture of the plantar fascia is a rare but potentially debilitating injury in athletes, particularly those involved in running and jumping sports. Early recognition and prompt treatment are crucial for a successful recovery and return to play. Conservative treatment, including rest, immobilization, and physical therapy, may be effective in most cases, while surgical intervention may be required in those that are nonresponsive to conservative treatment.

We report a case of plantar fascia rupture in a 22-year-old male semi-professional football player who presented with sudden severe pain in the sole of his right foot during a match, followed by a popping sensation and inability to weight bear. The athlete was healthy and had no history of previous injury in the right foot. MRI confirmed a complete rupture of the plantar fascia. The player was treated conservatively and underwent a rehabilitation program. The player returned to full competition after nine weeks, with no limitations.

## Introduction

The plantar fascia is a fibrous tissue that runs along the sole of the foot and helps support the arch. It is a crucial component of the foot anatomy, playing a key role in weight-bearing and stability during walking and other activities [1].

Despite its importance, the plantar fascia is also susceptible to injury, particularly in individuals who engage in repetitive high-impact activities, such as runners, jumpers, and other athletes who put repetitive stress on their feet [2]. Although plantar fasciitis is seen in athletes, tearing of the plantar fascia is a rare event that can occur spontaneously during physical activity [2,3].

Plantar fasciitis is a well-known disorder, and the most common complaint among patients is pain when taking the first steps in the morning [1]. In athletes, pain is often observed at the start of training. With the progression of the disease, pain may continue throughout the activity and may be refractory to treatment [4].

Plantar fascia rupture is not as common as plantar fasciitis. Plantar fascia rupture in athletes was first described by Leach et al. in 1978 [3], and most of the cases are known to occur in patients with prior plantar fasciitis [5]. Patients often describe an intense tearing sensation on the bottom of the foot. There is an association between previous injections of corticosteroids to treat plantar fasciitis and the increased risk of rupture [2,4].

The cases described in the literature are scarce and there is no consensus on the best therapeutic strategy to adopt. The baseline treatment is based on conservative therapy such as rest, analgesia, and physical therapy. Surgical treatment is recommended only if conservative treatment fails [6].

We describe a clinical case of a spontaneous acute rupture of the plantar fascia in a 22-year-old football player who was successfully diagnosed with MRI and treated with conservative measures, avoiding surgery.

## Case presentation

A 22-year-old male semi-professional center-back soccer player of the Portuguese Liga Revelação, with a right dominant leg, experienced sudden pain in his right rear foot as he landed after a jump during a soccer match. The athlete had never received steroid injections and had no history of previous plantar fasciitis.

He was evaluated on the pitch during the match and described a popping sensation and a sharp pain in the plantar region and heel. The athlete was incapable of weight-bearing on the right foot, presented an evident gait limitation, and was promptly removed from the field.

Physical examination revealed an abnormal alignment of the ankle-foot axis, with bilateral *cavus* and *varus* foot. There was local edema, ecchymosis, and pain on the palpation of the fascia and calcaneus.

Passive dorsiflexion of the metatarsophalangeal joint of the great toe also produced severe pain. Passive and active ranges of motion were preserved. There was no pain related to other structures of the foot, such as ligaments, tendons, or bone, and there was no anterior or posterior instability. An X-ray of the right foot was requested by the medical team on the same day, and the images did not reveal any signs of osseous lesions.

**Figure 1:**
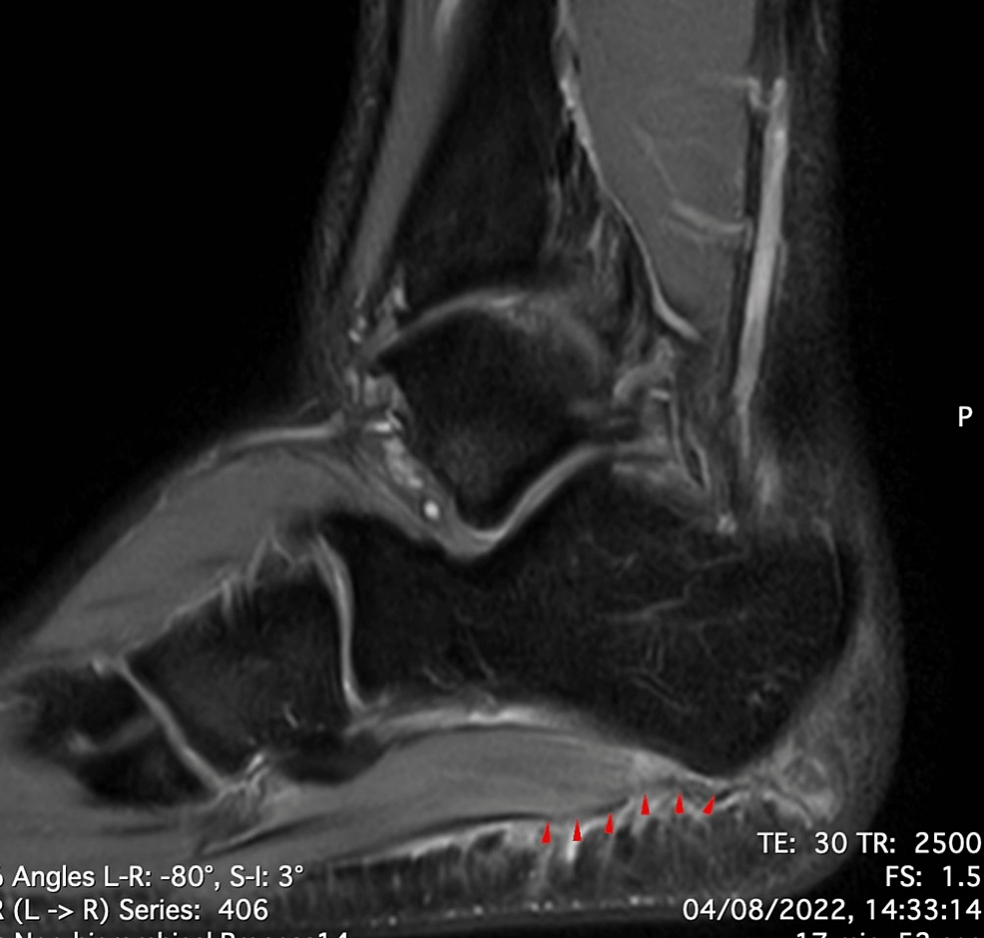
Saggital cut of proton-density MRI. Full-thickness tear of the proximal segment of the lateral band of the plantar fascia, which appears with a bright signal (red arrows).

**Figure 2:**
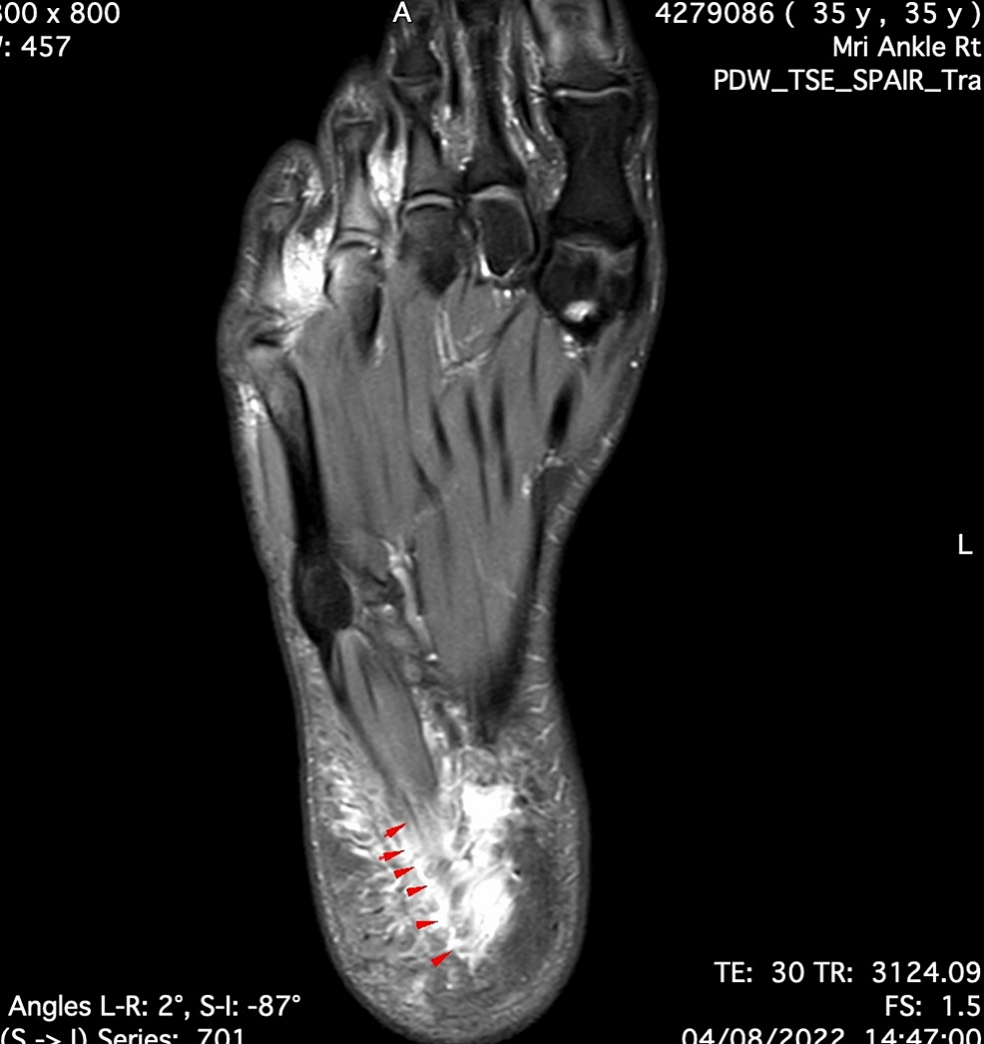
Axial cut of proton-density fat-saturated MRI. Tear of the proximal segment of the lateral band of the plantar fascia, which appears with a bright signal (red arrows). Note the edema in the surrounding tissues.

**Figure 3:**
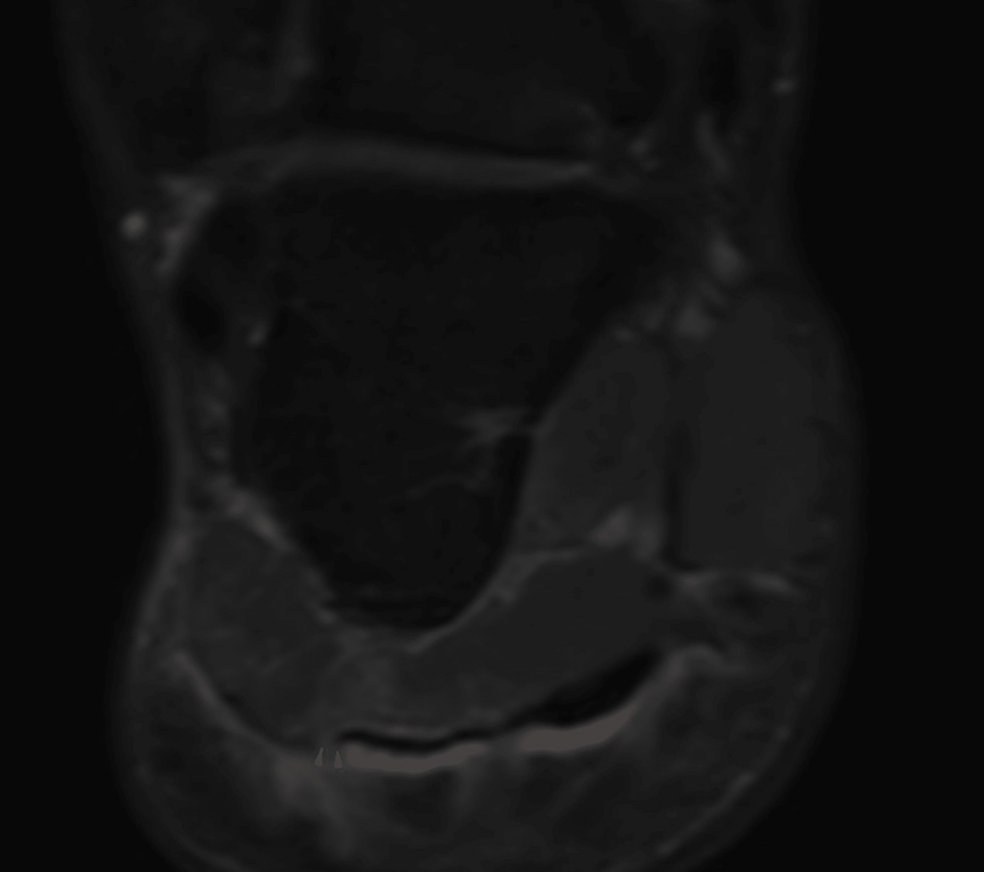
Coronal cut of proton-density MRI. Tear of the plantar fascia (red arrows).

Due to the persistence of symptoms three days after the injury, an MRI was requested. It revealed a rupture of the lateral fascicle of the plantar fascia with edema of the surrounding tissues (Figures 1-3).

Conservative treatment was initiated, with initial discharge and immobilization with a Walker boot for two weeks. Non-steroidal anti-inflammatory drugs (diclofenac 75 mg, two times per day) were used during the first three days, and cryotherapy and painkillers (paracetamol 1 g, three times per day) were prescribed during the first week.

The rehabilitation program began one day after the diagnosis. The athlete completed a one-hour session of physical therapy every day during the week. For analgesia, the treatment was transcutaneous electrical nerve stimulation at 100 Hz paresthesia-induced intensity and pulsed ultrasound with 20% duty cycle 3 MHz 1.2 w/cm^2^ to increase blood flow and reduce inflammation in the right rear foot.

A manual drainage massage was performed. A gentle and progressive stretch of peri-articular ankle muscles and intrinsic muscles of the foot was performed. Strengthening exercises to improve the stability and strength of the foot and ankle were progressively implemented, with a greater focus on eccentric work. Proprioceptive, balance, and motor coordination work was conducted from the early stages along with biomechanical analysis and correction of foot alignment. The final phase of the rehabilitation program consisted of plyometric and sport-specific exercises.

There was a favorable and progressive evolution, and the athlete returned to play nine weeks later, without any physical limitations.

## Discussion

Acute tears of the plantar fascia are rare injuries in athletes, especially if they occur spontaneously, as in the presented case [2,5,6]. Acute tears can be partial or complete [7].

Some risk factors predispose to this kind of injury. One mechanism appears to be due to an excessive load on a weakened structure. Most authors describe that previous fasciitis or local corticosteroid injection seem to injure the plantar fascia, increasing the probability of rupture [5,6,8]. In the described case, the patient did not have a history of any lesions or previous pain on the right foot. He also denied taking oral corticosteroids. The plantar fascia is an extensive, multilayered fibrous structure that acts by a windlass mechanism and plays an important role in the terminal stance phase of the gait cycle [8]. Because of this non-elastic property, the metatarsophalangeal joints are fully extended during push-off, shortening the length of the plantar fascia and increasing the strain in the medial longitudinal arch, making this area more vulnerable to lesions [8,9]. The athlete had pes* cavus* and *varus*. In high-arched feet (*pes cavus*), the strain in the fascia increases and has less mobility to absorb ground reaction forces, increasing the load applied to the foot. This condition has been shown to predispose to plantar fasciitis and plantar fascia rupture [10]. Another risk factor, not specific for fascia rupture, but for injuries in soccer is the excessive number of matches [11]. Many of the patient’s teammates had recently suffered injuries and the player had to play two games per week on average in the month of sustaining the injury. An excessive number of matches and the consequent load on the fascia seem to have been important risk factors for the rupture.

The diagnosis is mainly clinical based on medical history and physical examination which are important criteria to distinguish between acute or chronic rupture [5,6,8]. Although acute ruptures are often accompanied by a typical *pop sound* and a plantar hematoma, these signs are missing in acute-on-chronic ruptures [5]. The player presented a typical history of acute rupture with sudden sharp pain and a *pop* sensation in the sole of the foot after a sudden effort (landing in a jump), as well as local ecchymosis and the inability to fully bear weight. In addition to the clinical examination, imaging studies such as ultrasound or MRI should be used to confirm the diagnosis and assess the extent of the damage [7]. The typical sign on MRI is a complete or partial interruption of the low signal intensity plantar fascia, with a high T2/short tau inversion recovery signal. Ultrasound characteristics include the disruption of the plantar fascia and hypoechoic tissue in the region of the rupture resulting from inflammation/hemorrhage [6]. Some authors consider MRI to be the gold standard for diagnosis [7], while others report that ultrasound is superior to MRI in differentiating true fiber interruption and tearing from edema [12]. Radiographs are not used for diagnosis but are important for excluding concomitant fractures or dislocations [7].

Despite no guidelines or other recommendations for the treatment of this condition, most patients are treated conservatively, with surgery performed in only a few cases in the literature [5].

Conservative treatment is based on immobilization, boot brace, and limited weight-bearing [6]. Immobilization of the affected foot with a rigid walker boot is recommended for two weeks and pain-adapted weight-bearing should be allowed in the rigid walker [5]. Physical therapy was realized in most studies and has been proven to play an important role in the treatment [5,6]. Platelet-rich plasma and shockwave therapy were used in some cases. Although they are highly used in plantar fasciitis, their use is not defined in plantar fascia rupture [5]. Regarding the return to play, there is great variability in the studies, ranging from three to 64 weeks, with an average of three and a half months (14.7 weeks) [5]. However, our athlete was able to successfully return to a soccer game after nine weeks of conservative treatment.

## Conclusions

Plantar fasciitis is a common disease in athletes but acute plantar fascia rupture is a less common injury that should not be overlooked in the differential diagnosis. With this case report, we intend to raise awareness about the possibility of this pathology and emphasize the importance of the diagnosis based on the clinical history and physical examination and confirmed by imaging (MRI or ultrasound). In this case, recovery was complete with conservative treatment and the player was able to compete at his previous level. We hope that more cases will be reported in the future to facilitate the creation of a standardized treatment protocol.
